# Trainee Principal Investigator Could Improve Recruitment in Trauma Trials: Review of Literature and Experience From a Trauma Center

**DOI:** 10.7759/cureus.18920

**Published:** 2021-10-20

**Authors:** Aurelia Vas, Prashanth D'sa, Sandeep Gokhale, Tanvi Agarwal, Gareth L Roberts, Khitish Mohanty

**Affiliations:** 1 General Surgery, Princess of Wales Hospital, Bridgend, GBR; 2 Trauma and Orthopaedics, University Hospital of Wales, Cardiff, GBR; 3 Otolaryngology, Princess of Wales Hospital, Bridgend, GBR

**Keywords:** junior doctor, recruitment, trainee principal investigator, trauma trial, randomized control trial

## Abstract

Introduction: Recruitment of patients to participate in randomized control trials (RCT) is a challenging task, especially for trauma trials in which the identification and recruitment are time-limited. Multiple strategies have been tried to improve the participation of doctors and the recruitment of patients. The aim was to study the effect of a trainee principal investigator (TPI) on the efficacy of recruitment for a multicenter hip fracture RCT.

Methods: A retrospective study comparing the number of junior doctors participating in the WHiTE 8 COPAL RCT and patients recruited before and after the introduction of formal TPI role at a major trauma center in the UK. Data was collected for nine months “before” (Nov 2018-July 2019) and six months “after” (Sept 2019-Feb 2020) the role of TPI was assigned.

Results: From November 2018 to February 2020, a total of 292 patients were eligible for recruitment into this trial, out of which 196 (67.12 %) were successfully recruited. Excluding the research team, there were seven junior doctors actively recruiting in the “before period” in comparison with 10 in the “after period.” Significantly more patients were recruited by junior doctors after a TPI was assigned. Overall, more percentage of eligible patients were recruited into the trial after a TPI was assigned, and this was statistically significant.

Conclusion: The allocation of a formal TPI significantly improved the recruitment of patients in a national RCT. TPI can work alongside the principal investigator and research team to be a valuable link person coordinating and engaging local trainees to take part in trials.

## Introduction

Clinical trials are the cornerstone upon which modern evidence-based medicine has been built. Recruitment of patients to participate in randomized control trials (RCT) is known to be a challenging task [1,2], especially for trauma-related trials in which the identification and recruitment are time-critical. Due to this, many trials fail to reach target recruitment, resulting in either underpowered studies, jeopardizing the validity of the results, or require timeline extensions incurring increased costs, or are prematurely closed [3-7]. Between 2002 and 2008, only about 55% of the National Institute for Health Research, UK (NIHR)-funded RCTs managed to meet their recruitment targets in time, and others had to be extended [3].

Clinician-related factors have been cited as one of the main reasons for low rates of recruitment in trials across different care settings [8]. Multiple strategies have been tried to improve the participation of doctors, nurses, and the recruitment of patients [9]. Some methods used to improve staff participation include digital nudging, telephone reminders, site champion, associate/trainee principal investigator (TPI), improving staff training, and financial incentives [7,10-16]. Results from multiple studies evaluating the effect of these interventions have been mixed [11,12,14,15].

Taking part in research activities is both essential and relevant to junior doctors in training, as per standards set by General Medical Council’s “Capabilities in research and scholarship” (Domain 9, Generic professional capabilities framework [GMC], UK) and in the certification guidelines set by Joint Committee on Surgical Training (JCST), UK [17,18]. Junior doctors in training as a whole have the potential to become the largest recruiting force to aid the trials to reach their planned targets in time. There are multiple barriers that junior doctors face to take part in trials including unfamiliarity, lack of time, lack of rewards and recognition, lack of training, difficulty with consent procedure, and worry about the doctor-patient relationship [8,19]. Having a junior doctor who could guide/nudge their peers on the process of getting involved in research trials, coordinate patient recruitment, could improve the overall efficiency, thus reducing the burden on research staff. The TPI’s role has been mentioned to be used in orthopedic RCTs by many trial units [16], however, there has been no published literature looking at the effect of this intervention on recruitment.

We hypothesized that a TPI could improve participation by junior doctors and improve overall recruitment for a multicenter hip fracture RCT at a major trauma center.

This article was previously presented as a short free paper at the 2021 SICOT Orthopaedic World Congress on September 17, 2021.

## Materials and methods

This is a retrospective analysis of prospectively collected data, looking at the efficacy of recruiting eligible patients to WHiTE 8 COPAL RCT before, and after having a TPI at a major trauma center in the UK. The WHiTE 8 COPAL Or Palacos antibiotic-loaded bone cement trial is a pragmatic, multicenter, multi-surgeon, parallel, two-arm, randomized clinical trial of low-dose single antibiotic-loaded bone cement versus high-dose dual antibiotic-loaded bone cement in patients receiving a hip hemiarthroplasty after fracture [20]. The eligibility of patients for the trial was defined by the WHiTE 8 COPAL study protocol [20]. We also performed a comparative analysis of the number of junior doctors actively involved in recruitment, and whether the recruitment target was achieved. The recruitment target set by the central trial unit was nine patients per month. Our site was registered for recruitment for the trial in October 2018, and the first patient to be recruited by a junior doctor was in November 2018. A TPI among the junior doctors was assigned by the local principal investigator (PI) in September 2019. A trainee is a junior doctor in any type of surgical training program/residency. The role of TPI included recruiting eligible patients for the trial, support other junior doctors in the department through the process of enrolling into the trial, organizing training sessions in the recruitment process, and overall coordination of the recruitment process alongside the research team. The research nurses and research manager make up the research team, and, they help the clinicians in recruitment, coordination of various trials, organize training sessions for those interested in taking part in trials, and collect follow-up data for trial patients. The “Pre TPI” period was November 2018 to July 2019 and the “Post TPI” period was from September 2019 to February 2020. Data from August 2019 was excluded because, there was no trainee on the delegation log, as the new trainee rotation starts in August every year. Recruitment for the trial stopped on 23rd February 2020 due to the COVID-19 pandemic. This study has been registered and approved by the local research department as a service evaluation.

The Statistical Product and Service Solutions package (IBM SPSS Statistics for Windows, Armonk, New York, USA) was used for all statistical analyses. We used the chi-square test to check for the statistical significance of the 2×2 contingency tables. A p-value of less than 0.05 was considered significant.

## Results

From November 2018 to February 2020, a total of 292 patients were eligible for recruitment into the WHiTE 8 COPAL trial, out of which, 196 (67.12 %) were successfully recruited. The number of patients eligible, recruited, missed, and declined participation across both periods have been shown in Table 1. Excluding the research team, there were seven trainee junior doctors actively recruiting in the before period, in comparison with 10 in the after period. Significantly more patients were recruited by junior doctors after a TPI was assigned (see Table 2).

**Table 1:** Number of patients eligible, recruited, missed, and declined for participation in WHiTE 8 COPAL trial before and after having a TPI TPI: trainee principal investigator

	Pre-TPI period	Post TPI period
Eligible	157	135
Total recruited	94 (60%)	102 (76%)
Recruited by junior doctor	35 (37%)	72 (71%)
Recruited by research team	59 (63%)	30 (29%)
Missed	52 (33%)	29 (21%)
Patient declined	11 (7%)	4 (3%)

**Table 2:** Number of patients recruited by junior doctors before and after having a TPI. TPI: trainee principal investigator; JD: junior doctors; RT: research team

	Recruited by JD	Recruited by RT	P Value
Pre TPI period	35 (36%)	59 (64%)	X^2^ (2, N = 196) = 21.95, P = < 0.001
Post TPI period	72 (71%)	30 (29%)	

**Figure 1:**
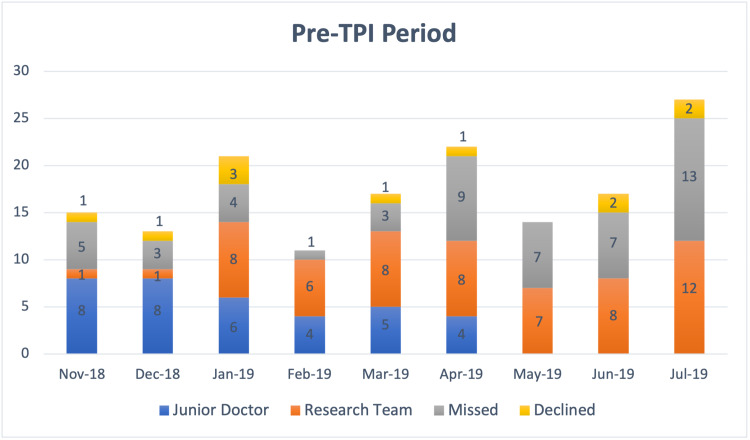
Patients recruited by junior doctors, research team, missed and declined for recruitment in the “Pre TPI” period of November 2018 to July 2019 TPI: trainee principal investigator

**Figure 2:**
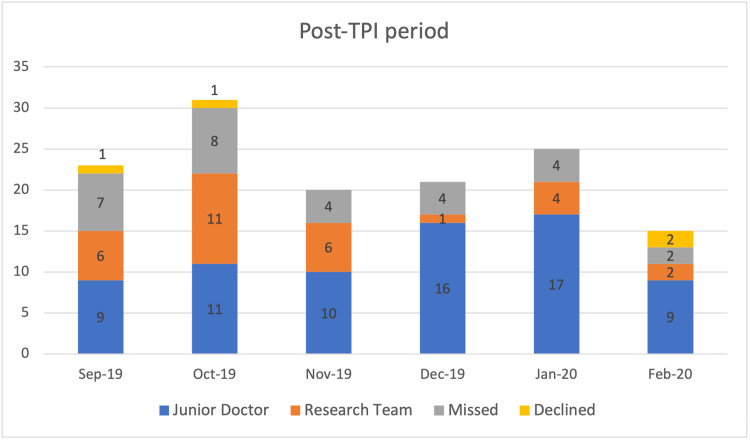
Patients recruited by junior doctors, research team, missed and declined for recruitment in “Post TPI” period of September 2019 to February 2020 TPI: trainee principal investigator

Overall, more percentage of eligible patients were recruited into the trial after a TPI was assigned (see Table 3). This was statistically significant. The monthly target for recruitment was missed in 2 months prior to the assignment of TPI, whereas, the target was achieved in all the months after TPI was assigned. Monthly recruitment figures of patients recruited by junior doctors, research team, missed, and declined for recruitment in both periods are detailed in Figures 1, 2.

**Table 3:** Number of patients recruited before and after having a TPI TPI: trainee principal investigator #Patients not recruited includes eligible patients who were missed and who declined to participate

	Successfully Recruited	Not Recruited ^#^	P Value
Pre TPI period	94 (60%)	63 (40%)	X^2^ (2, N=292) = 8.09, P = 0.0044
Post TPI period	102 (76%)	33 ^(^24%)	

## Discussion

There is a paucity of evidence evaluating strategies aimed at recruiters for improving participation and recruitment to trials. Cochrane systematic review and meta-analysis by Treweek et al. [9] assessed the effectiveness of 46 different interventions on recruitment to RCTs in 45 trials. Majority of the included studies (n=40) evaluated interventions targeted toward trial participants, with only a few studies (n=5) evaluating the interventions on recruiters. The interventions that were found to be effective by the review were open trial design, telephone reminders to patients to follow-up, written invitations, use of opt-out approach, and financial incentives to patients to encourage participation. Strategies targeted toward recruiters such as educational packages, increased trial center coordination through on-site visits, and additional communication did not show a significant impact on recruitment.

A survey of central trial units in the UK conducted by Bower et al. [21] investigated various interventions used by them to improve recruitment and retention in trials. Some of the methods used by these units aimed at recruiters were support for recruiters, recruitment targets, competition among sites, gifts, financial incentives, co-authorship, face-to-face initiation, regular contact with site staff, and site champions. They identified the top priorities for further evaluation which are training site staff, methods of communication with patients, and incentives for patients/site staff.

TPIs are currently used in different trials in many centers across the UK in orthopedic trials with some anecdotal evidence of recruitment improvement [16]. However, their effect on recruitment has not been evaluated, and a literature search on PubMed and Google Scholar revealed the absence of any published studies on this subject. A large multicenter RCT recently published its protocol for a factorial RCT embedded within its original trial to evaluate the effect of enhanced TPI package and additional digital nudge to improve recruitment rates, the results of which are awaited [16]. NIHR is currently running an associate PI scheme for multiple trials across different subspecialties endorsed by the Royal colleges and is available for junior doctors, nurses, and allied health professionals for taking part [22].

The local PI at our center selected a TPI among the trainee junior doctors to engage their colleagues in the department to take part in the RCT by helping them overcome their barriers about participation due to poor participation in the first month of the rotation. Our center was not a part of the factorial RCT, study protocol of which was recently published, and we were not aware of such planned interventions at the time of selection of the TPI locally [16]. Apart from recruiting eligible patients for the trial, the TPI supported their colleague junior doctors, through the process of enrolling into the trial and organizing training sessions on the recruitment process. TPI also coordinated the recruitment process along with the research team by using an encrypted social media chat group, which also provided an opportunity for any queries/doubts the other recruiters raised to be answered promptly. When compared to the research team which largely consists of non-clinical staff or PI who is usually a senior consultant, it is easier for the TPI to gently nudge a colleague junior doctor whom they work with regularly to participate in trials and patient recruitment. Recruiting patients to RCTs is one of the alternate requirements for research competence fulfillment for completion of certified training (CCT) in trauma and orthopedics as per JCST [18]. The role of TPI involves managing and teaching other junior doctors, hence this enables trainees to fulfill management aspects in their portfolio. This role could also potentially help as a stepping stone toward future leadership roles in large research projects. Though currently not a part of, but has the potential to be considered as an alternate requirement of “Advanced research evidence” in CCT criteria in par with other mentioned managerial roles in research. The drawbacks of this role, however, include a trainee junior doctor that rotates to a different center in the middle of the recruitment period of the trial, which could potentially disrupt this arrangement until another suitable trainee junior doctor is selected.

Sharing monthly newsletters from the central trial unit as well as local leader board positions had created a feeling of some level of competition among the recruiters. This has resulted in most trainees continuing to engage in recruitment beyond the minimum numbers for collaborative authorship/certification requirements (n=5 for RCT) being met. In comparison, it may be noted (Figure 1) that in the last three months of the pre-TPI study period the recruitment figures by junior doctors reached zero after trailing, and the most likely reason for this could be a lack of motivation among junior doctors to recruit any further after they have reached the minimum required number for gaining recognition for participation in trials.

Limitations of this study include its retrospective design with its inherent selection bias and do not include potential confounding variables. Apart from introducing a TPI, no other changes were made in the department to improve recruitment, however, time-related changes in recruitment trends cannot be ruled out here and is a limitation of this review. The study looks at data from a single center and is of short duration with relatively small numbers. This intervention also needs a motivated trainee working alongside the research team to take up the role which itself is a challenge and could be a bias on its own as this could influence overall recruitment and may not be applicable in all trials. A large multicenter prospective trial should hopefully be able to provide further evidence on this intervention. To the best of our knowledge, this is the first study that formally evaluates the effect of a trainee junior doctor assuming the role of associate PI to improve recruitment of their peers to take part in RCTs, provide guidance, and coordinate overall recruitment for a trauma trial. The authors suggest that the TPI role should be formalized for all trials where trainees could take part in recruitment, and consider including them in authorship in all resulting publications from the trial in par with PI. This will act as further incentive for this role and aid in portfolio-building for trainees.

## Conclusions

The allocation of a formal TPI significantly improved the recruitment of patients in a national RCT. TPI can work alongside the PI and research team to be a valuable link person coordinating and engaging local trainees to take part in trials. This may be particularly beneficial in hospitals where there is no dedicated research team. TPI role could be formalized for many trials and can be used as a leadership and management potential building experience for trainees. Further research is needed on this subject to evaluate its effectiveness.
